# Efficacy of Corneal Collagen Cross-Linking for Treatment of Keratoconus: A Meta-Analysis of Randomized Controlled Trials

**DOI:** 10.1371/journal.pone.0127079

**Published:** 2015-05-18

**Authors:** Jingjing Li, Peng Ji, Xiaoti Lin

**Affiliations:** 1 Department of Ophthalmology, Xiangyang Central Hospital, Teaching Hospital of Medical College of Hubei University of arts and science, Xiangyang, Hubei Province, China; 2 Department of Operating Theatre, Xiangyang Hospital of Traditional Chinese Medicine, Teaching Hospital of Hubei University of Chinese Medicine, Xiangyang, Hubei Province, China; 3 Department of Surgery, Fujian Provincial Tumor Hospital, Teaching Hospital of Fujian Medical University, Fuzhou, China; Casey Eye Institute, UNITED STATES

## Abstract

**Objective:**

To evaluate the efficacy of corneal collagen cross-linking (CXL) for the treatment of keratoconus.

**Methods:**

We performed a literature search for randomized controlled trials that assessed the effect of CXL in slowing progression of keratoconus. The primary outcome measures included changes of topographic parameters, visual acuity, and refraction. Efficacy estimates were evaluated by weighted mean difference (WMD) and 95% confidence interval (CI) for absolute changes of the interested outcomes.

**Results:**

Significant decrease in mean keratometry value, maximum keratometry value and minimum keratometry value were demonstrated in the CXL group compared with the control group (WMD = -1.65; 95% CI: -2.51 to -0.80; P < 0.00001; WMD = -2.05; 95% CI: -3.10 to -1.00; P < 0.00001; WMD = -1.94; 95% CI: -2.63 to -1.26; P < 0.00001; respectively). Best spectacle-corrected visual acuity improved significantly in CXL group (WMD = -0.10; 95% CI: -0.15 to -0.05; P < 0.00001), whereas uncorrected visual acuity did not differ statistically. Manifest cylinder error decreased significantly in patients undergoing CXL procedure compared with control patients in sensitivity analysis (WMD = -0.388; 95% CI: -0.757 to -0. 019; P = 0.04). The changes in central corneal thickness and intraocular pressure were not statistically significant.

**Conclusion:**

CXL may be an effective option in stabilizing keratoconus. Further long-term follow-up studies will be necessary to assess the persistence of CXL.

## Introduction

Keratoconus is the commonest corneal degenerative disorder characterized by para-central corneal thinning and secondary conical ectasia, resulting in irregular astigmatism and progressive myopia or visual loss [1]. It affects approximately one in 2000 in the general population [2]. Moreover, the quality of life researches reveal that its degree of public health impact is disproportionate to its incidence, and almost equal to grade three to four age related macular degeneration [3]. Finally, about 20% keratoconic cases require corneal transplantation to restore vision [4].

Corneal collagen cross-linking (CXL) using ultraviolet A (UVA) and riboflavin is a promising treatment to slow or halt the progression of keratoconus [5]. No intervention was available to prevent or stop progression of the disease before the advent of CXL. This novel technology with a combination of riboflavin (vitamin B2) eye drops to be absorbed throughout the cornea stroma and UV-A radiation which triggers photochemical reaction to change the cross links between and within collagen fibers may increase the biomechanical stiffness of the corneal stroma [6–8]. In 2003 Wollensak et al firstly reported its use in keratoconic eyes [5]. After that more and more non-randomized prospective and retrospective studies published the encouraging outcomes in advanced keratoconus throughout the world [9–12], including two meta-analysis which assessed the efficacy of CXL [13,14]. However, majority of these researches as well as the two meta-analysis comparing preoperative and postoperative outcomes had no control group. Until recently, there is only a scarcity of randomized controlled trials (RCTs) of CXL in keratoconus within the literature [15–20]. In order to provide powerful evidence for the widespread clinical practice of this new therapeutic method, we undertook this meta-analysis of all published RCTs to evaluate the effect of CXL in the treatment of keratoconus. To our knowledge, this is the first study to pool the important outcome measures from RCTs of epithelium-off CXL in keratoconus.

## Materials and Methods

### Search strategy

We performed the present meta-analysis in accordance with the Meta-Analyses statement [21]. PUBMED, EMBASE, ISI Web of Science, ClinicalTrials.gov, and the Cochrane Central Register of Controlled Trials were searched for RCTs that assessed the effect of CXL on keratoconus in humans. And we conducted the searches for publications from year 2000 to 31 September 2014. It was identified that the first published article evaluating the effect of CXL in patients with keratoconus was in 2003 [5], so the year 2000 as the starting point for the literature search was reasonable. Two authors (JJL and PJ) independently conducted a systematic literature search by using the following key words: “cross linking”, “crosslinking”, “cross—linkage”, “cross-linking”, “keratoconus”, and “keratoectasia” with no language restrictions. The Medical Subject Heading (MeSH) terms of “cross-linking reagents” and “keratoconus” were also searched. In addition, we performed a manual search of the bibliographies of retrieved articles.

### Inclusion and exclusion criteria

Studies were considered selected if they met the following criteria: 1) study design: randomized controlled trial; 2) population: patients 14 years of age or older, with a confirmed diagnosis of keratoconus, or documented progression of the disease; 3) intervention: standard UVA—riboflavin 0.1% CXL treatment vs. control group; Epithelium-off CXL was conducted according to a modification of the Dresden protocol [8].4) outcome variables: the end points of interest were changes in topographic parameters, distance visual acuity, refraction. We initially scrutinized the titles and abstracts of all electronic references, and then rescreened full-text articles.

### Outcome measures

Between baseline and endpoint visits, the following outcome measures were analysed: distance uncorrected visual acuity (UCVA) (expressed in logarithm of the minimum angle of resolution [LogMAR] units), distance best spectacle-corrected visual acuity (BSCVA) (expressed in LogMAR units), subjective refraction (spherical, cylindrical error, and spherical equivalent), maximum keratometry value (K_max_), minimum keratometry value (K_min_), average keratometry value, (or mean keratometry value, K_mean_), central corneal thickness (CCT) and intraocular pressure (IOP). Efficacy was determined as the absolute changes of these outcome measures from baseline to endpoint. The primary outcome of interest was reduction in topographic measurements. Changes of visual acuity, refractive error, CCT and IOP were also investigated as secondary outcomes.

### Data extraction

Two authors (J.J.L. and P.J.) evaluated the quality of the citations and performed data extraction independently. Any disagreements were reconciled by discussion. We extracted the data using a standard data-collection form. Data were recorded as follows: author name, year of publication, location of the trial, study design, study duration, numbers of eyes, mean age, sex, ocular parameters between baseline and different follow-up time points. The decimal scale of visual acuity was converted into the LogMAR scale when we extracted the data.

### Quality assessment

The quality of included trials were assessed with Jadad scoring system for RCTs [22]. The Jadad instrument evaluates randomization, generation of random numbers, double blinding (participant masking and researcher masking), the description of withdrawals and dropouts, as these are inherent controls of bias. Total scores ranged from 0 (poor quality) to 5 (excellent quality). Allocation concealment was also considered [23]. Disagreements in ratings were resolved by negotiation between the two authors (J.J.L. and P.J.).

### Statistical analysis

Analyses were carried out using STATA version 12.0 (StataCorp LP, College Station, Texas). Treatment effects were evaluated as weighted mean difference (WMD) and 95% confidence interval (CI) calculated for absolute changes of the interested outcomes. For individual articles, WMD was computed by the difference of the mean change in the CXL group and that in the control group. The outcomes were measured as mean ± standard deviation (SD). Heterogeneity across studies was estimated by using chi-square test, which believed to be statistically significant if P < 0.10. The quantity I^2^ statistic was also calculated (I^2^ > 50% indicating significant heterogeneity). Additionally, we conducted a sensitivity analysis excluding study of poorer quality (Jadad scores < 3) when we pooled the data of manifest cylindrical error. We used a random effect model if significant heterogeneity was existed among trials. Alternatively, results were combined using a fixed effect model.

When a SD of an outcome change was not directly available, it was calculated from standard error of the mean (SEM), 95% CI, P value, or t value [24]. Potential publication bias was examined by Begg rank correlation test and Egger linear regression test when there were sufficient studies in final analysis, otherwise, we did not conducted the test. A P value < 0.05 was considered to be statistically significant, except where otherwise specified.

## Results

### Characteristics of trials

**Fig 1:**
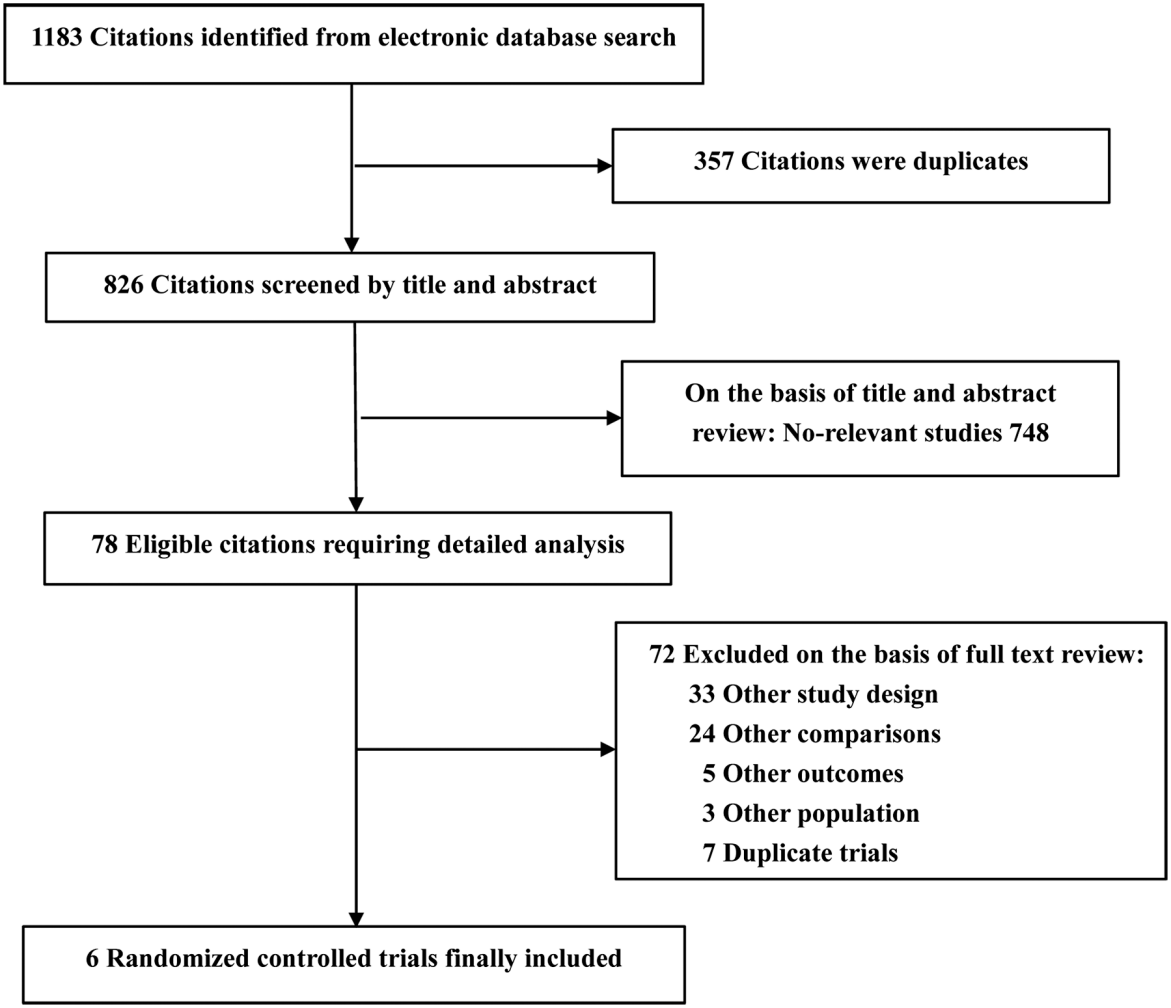
Flow chart of studies included in the meta-analysis.

Detailed processes of the related study selection are presented in Fig 1. Finally, a total of six RCTs fulfilling the eligibility criteria were retained for the meta-analysis [15–20]. There were 179 eyes included in the CXL group, 182 eyes included in the control group. The control group of two studies received a sham treatment [16,18]. In sham control group, riboflavin 0.1% eye drops were administered alone. After three months, one group underwent insertion of intrastromal corneal ring segments [16], another sham control group crossed over to the treatment group and received full CXL treatment [18]. The later study also had a fellow-eye control group [18]. We only pooled the data before the change of treatment which was comparable. Moreover, a trial conducted by Hersh et al was a multicenter clinical study [18]. Two studies by Wittig-Silva were separate studies at different time [15,20]. These researches were reported between 2008 and 2014. Duration of follow-up ranged from three months to 36 months. The characteristics of each trial and Jadad score are presented in Table 1. Among the included trials, five trials described use of random-number generation [15–18,20], and three trials reported adequate allocation concealment [15,17,18]. Furthermore, all of those trials revealed the number and reason of withdrawls or dropouts [15–20], and investigators were blind in two studies [16,17].

**Table 1:** Characteristics of 6 randomized controlled trials included in analysis.

Author	Year	Country	No. eyes*	Mean age (years)	Men (%)	Duration (months)	Baseline K_max_ (D)*	Jadad score
Wittig-Silva et al. [20]	2008	Australia	33/33	26.6	51.5	12	52.70±4.50/50.80±4.30	2
Henriquez et al. [19]	2011	Peru	10/10	29.7	80.0	12	47.45±4.13/48.62±3.61	2
Hersh et al. [18]	2011	USA	49/28/21^†^	≥14	NR	12	60.4±9.99^‡^	4
O'Brart et al. [17]	2011	UK	22/22	29.6	79.2	18	53.9/52.8	4
Renesto Ada et al. [16]	2012	Brazil	19/20	29.4	25.8	3	53.26±5.11/52.17±3.89	3
Wittig-Silva et al. [15]	2014	Australia	46/48	25.7	57.4	36	52.87±4.31/51.18±4.03	4

K_max_ = maximum keratometry value; D = diopters; NR: Not reported.

*Corneal collagen cross-linking group/Control group;

^†^Corneal collagen cross-linking group/Sham control group/Fellow-Eye control group;

^‡^Without baseline control group records.

### Topographic results

**Fig 2:**
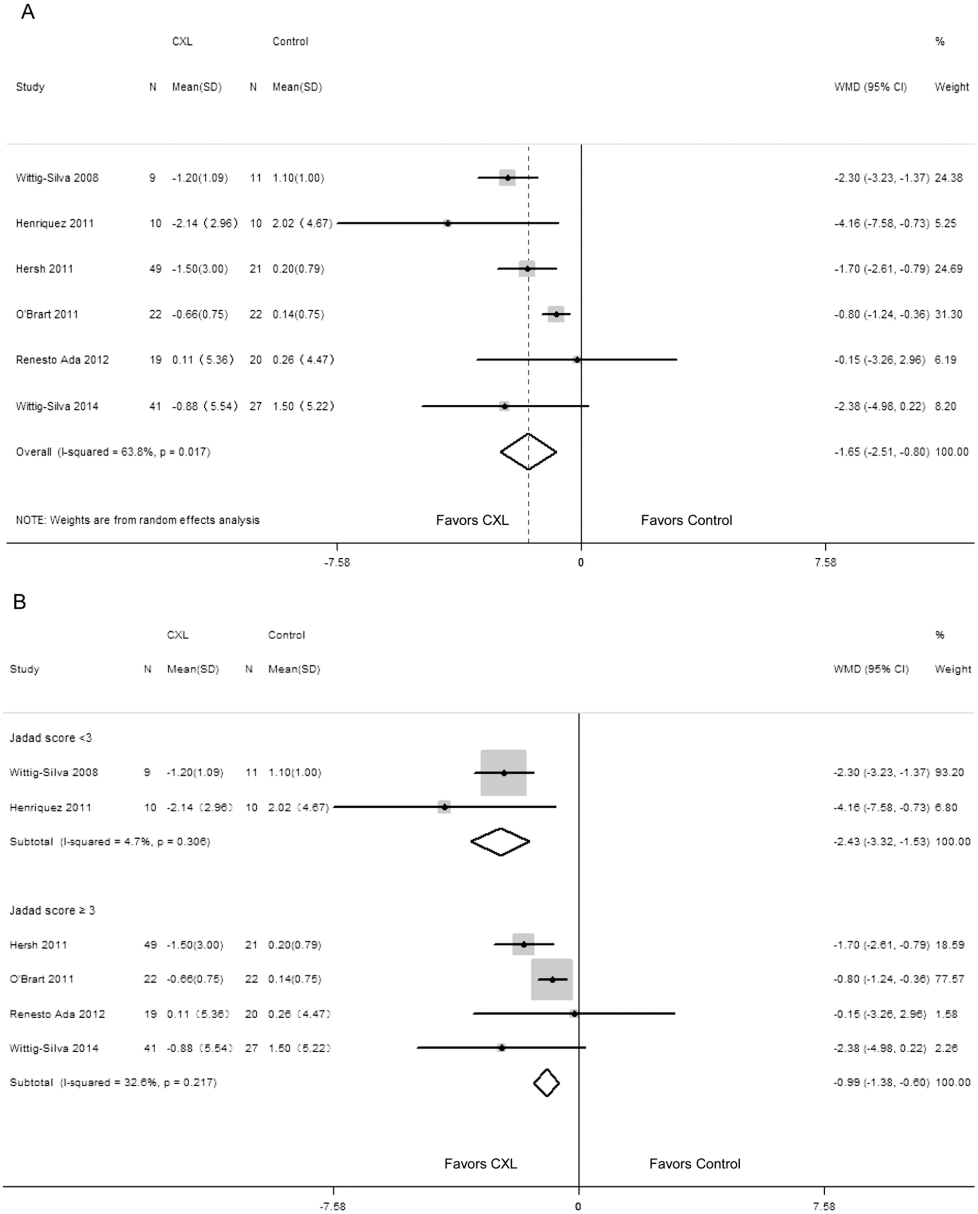
Change in mean keratometry value (diopters) between corneal collagen cross-linking (CXL) and control patients: overall change (A) and subgroup analysis according to Jadad score (B). WMD, weighted mean difference.

Forest plots illustrating changes in the K_mean_ between the CXL group and control group are provided in Fig 2. There was significant decrease in K_mean_ in the CXL group compared with the control group (WMD = -1.65; 95% CI: -2.51 to -0.80; P < 0.00001) (Fig 2A). But significant heterogeneity was present (P = 0.017, I^2^ = 63.8%). After conducting subgroup analyses according to Jadad score, significant improvement in K_mean_ was also demonstrated in the CXL group (WMD = -2.43; 95% CI: -3.32 to -1.53; P < 0.00001; WMD = -0.99; 95% CI: -1.38 to -0.60; P < 0.00001; respectively) (Fig 2B). No heterogeneity was found (P = 0.306, I^2^ = 4.7%; P = 0.217, I^2^ = 32.6%; respectively). Meanwhile, no evidence of publication bias was identified by using Begg rank correlation test (P = 0.707) and Egger linear regression test (P = 0.207).

**Fig 3:**
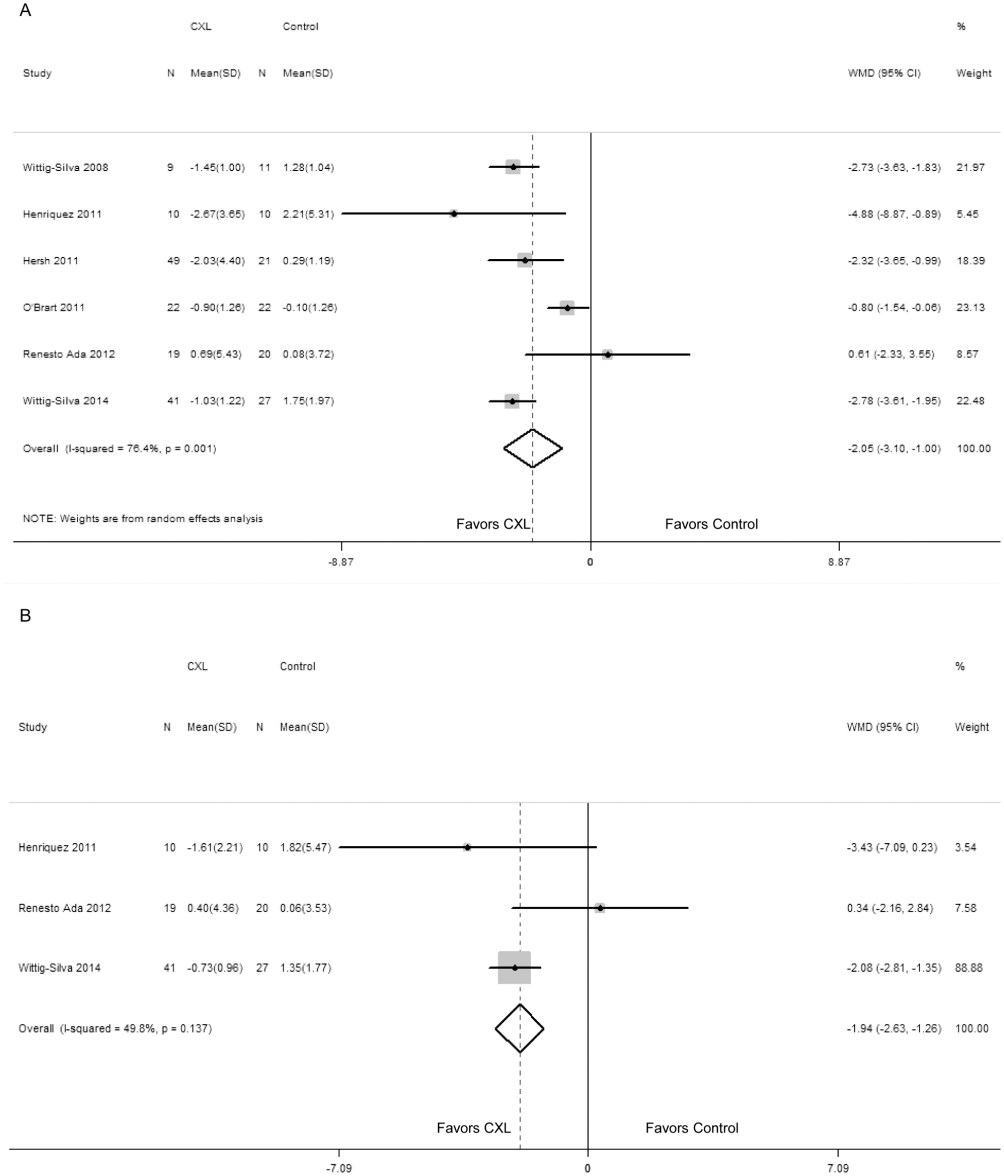
Change in maximum keratometry value (diopters) between corneal collagen cross-linking (CXL) and control patients (A); Change in minimum keratometry value (diopters) between CXL and control patients (B). WMD, weighted mean difference.

In addition, changes in K_max_ and K_min_ are shown in Fig 3. There was statistically significant evidence that K_max_ had changed (WMD = -2.05; 95% CI: -3.10 to -1.00; P < 0.00001) (Fig 3A), and heterogeneity was revealed (P = 0.001, I^2^ = 76.4%). Moreover, the decreases in K_min_ between the two groups were statistically significant (WMD = -1.94; 95% CI: -2.63 to -1.26; P < 0.00001) (Fig 3B). Heterogeneity was not shown (P = 0.137, I^2^ = 49.8%). Begg rank correlation test (P = 1.000) and Egger linear regression test (P = 0.838) did not show any publication bias.

### Visual acuity and refractive outcomes

**Fig 4:**
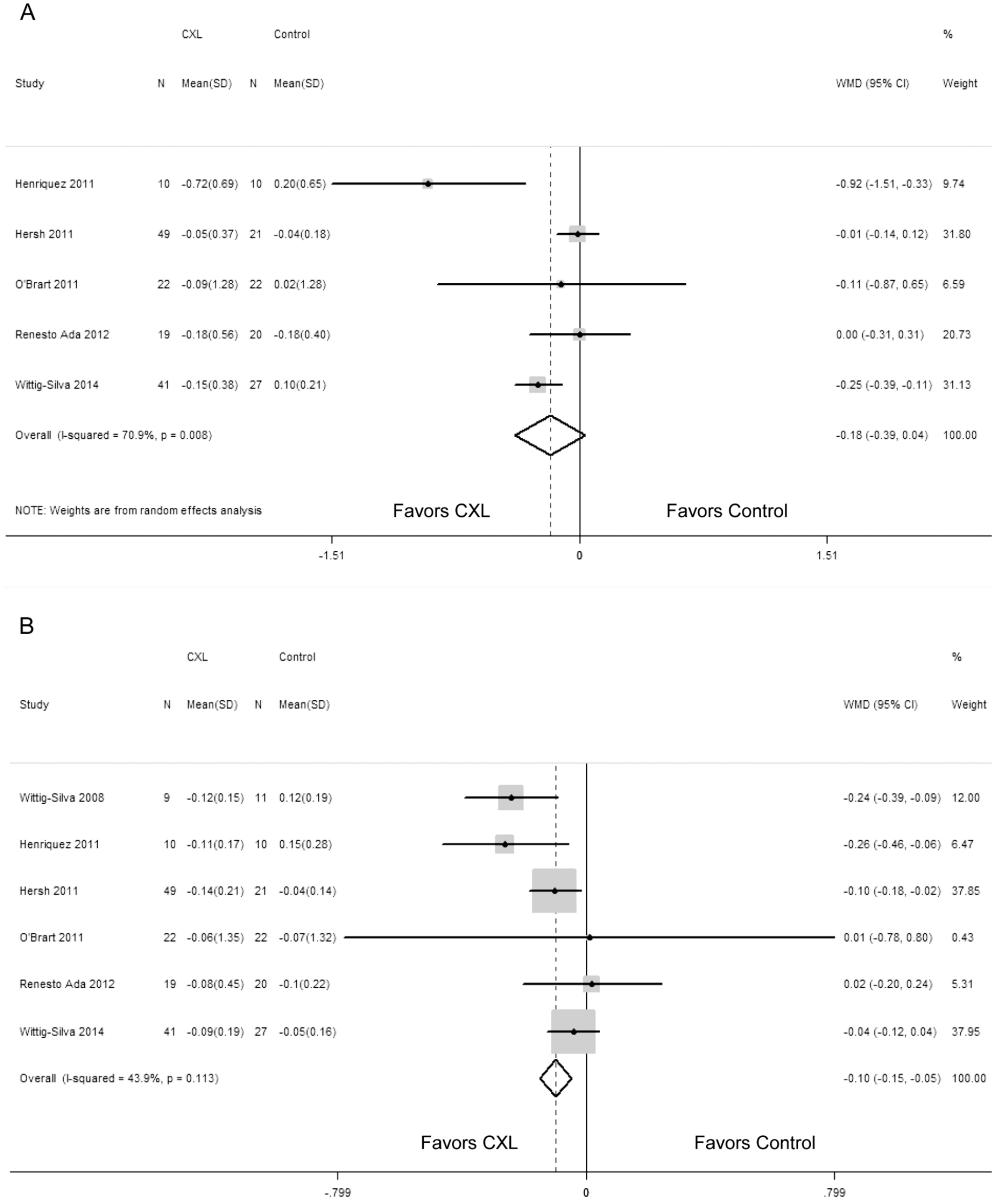
Change in uncorrected visual acuity (LogMAR) between corneal collagen cross-linking (CXL) and control patients (A); Change in best spectacle-corrected visual acuity (LogMAR) between CXL and control patients (B). WMD, weighted mean difference.

Compared with the control group, the UCVA was not significantly different in CXL group (WMD = -0.18; 95% CI: -0.39 to 0.04; P = 0.105) (Fig 4A), while heterogeneity was observed there (P = 0.008, I^2^ = 70.9%). Both Begg rank correlation test (P = 0.462) and Egger linear regression test (P = 0.529) did not demonstrate any publication bias.

However, treated eyes in CXL group significantly improved in BSCVA in comparison with the control group (WMD = -0.10; 95% CI: -0.15 to -0.05; P < 0.00001) (Fig 4B). No statistically significant heterogeneity between studies was identified (P = 0.113, I^2^ = 43.9%). And there was no significant publication bias by Begg rank correlation test (P = 1.000) and Egger linear regression test (P = 0.651).

**Fig 5:**
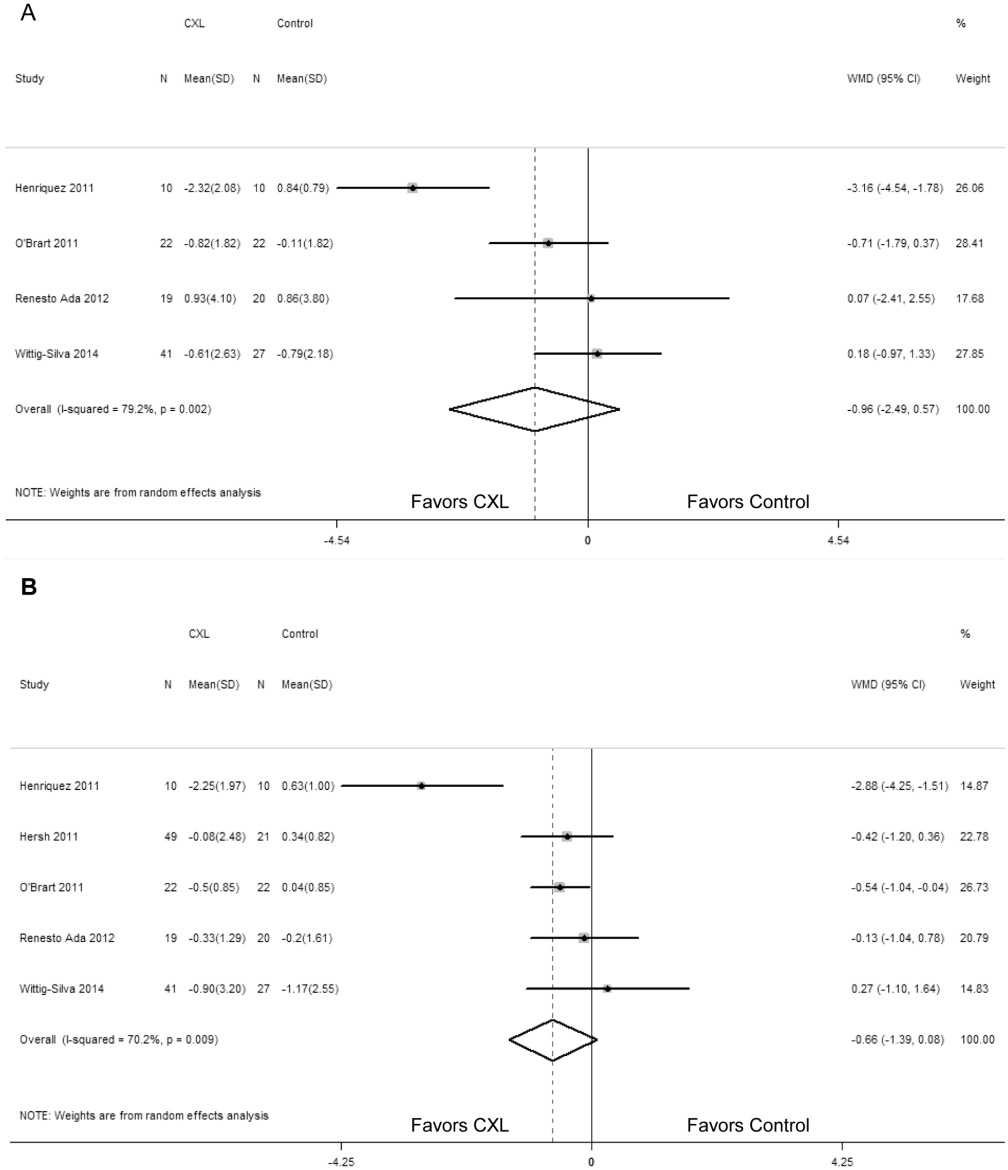
Change in spherical equivalent (diopters) (A) and manifest cylindrical error (diopters) (B) between corneal collagen cross-linking (CXL) and control patients. WMD, weighted mean difference.

The changes in the spherical equivalent and manifest cylindrical error did not differ significantly between the two groups (WMD = -0.96; 95% CI: -2.49 to 0.57; P = 0.218; WMD = -0.66; 95% CI: -1.39 to 0.08; P = 0.082) (Fig 5A and 5B). The test suggested heterogeneity in the two outcomes (P = 0.002, I^2^ = 79.2%; P = 0.009, I^2^ = 70.2%). With regard to the manifest cylindrical error, we performed a sensitivity analysis by excluding the study of poor quality. When we removed the study by Henriquez et al [19], it decreased significantly in patients undergoing CXL procedure compared with control patients (WMD = -0.388; 95% CI: -0.757 to -0.019; P = 0.04) and no heterogeneity existed (P = 0.671, I^2^ = 0). Begg rank correlation test (P = 0.462) and Egger linear regression test (P = 0.658) also did not revealed any publication bias.

### Central corneal thickness and intraocular pressure

**Fig 6:**
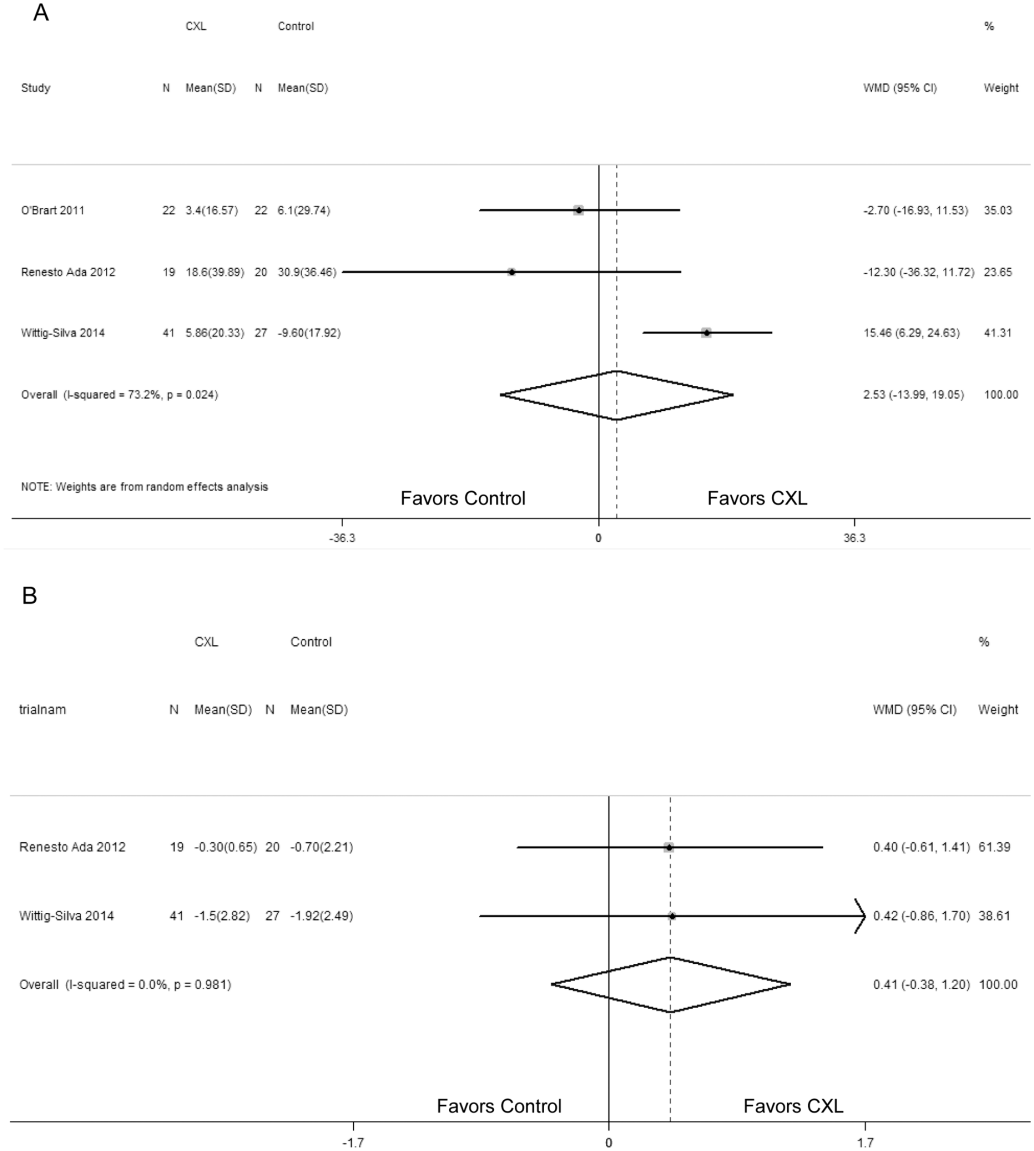
Change in central corneal thickness (um) (A) and intraocular pressure (mmHg) (B) between corneal collagen cross-linking (CXL) and control patients. WMD, weighted mean difference.

And what’s more, we pooled the data of CCT and IOP (Fig 6A and 6B). The analysis of these data indicated that the changes in CCT and IOP between the two groups were not statistically significant (WMD = 2.53; 95% CI: -13.99 to 19.05; P = 0.767; WMD = 0.41; 95% CI: -0.38 to -1.20; P = 0.313; respectively). No statistically significant heterogeneity between studies was identified in comparison of IOP (P = 0.981, I^2^ = 0), but existed in CCT (P = 0.024, I^2^ = 73.2%).

## Discussion

Our meta-analysis showed that CXL was effective in stabilizing keratoconus. The results demonstrated statistically significant improvement in K_mean_, K_max_, K_min_, BSCVA and manifest cylinder error in CXL treated group. A large number of published clinical trials had revealed a therapeutic effect of CXL in keratoconus. However, these studies could only provide limited level of evidence because of the lack of control data or non-random sampling. Hence, this analysis of 6 trials including a large groups of patients strengthen the power to offer more reliable assess. Meanwhile, each primary trial used a randomized controlled trial design, which greatly decreased the likelihood of confounders.

In our results, we found significant reductions in K_max_, K_min_, K_mean_ after CXL procedure. The improvement of K_max_ was demonstrated -2.05 D in the CXL group compared with the control group. Similar findings were reported in previous studies. Wollensak et al documented a reduction of the maximal keratometry value by 2.01 D [5]. Arbelaez et al reported decrease in the average keratometry reading of 1.36 D at 12 months, which was consisted with our data of 1.65 D [12]. Moreover, Henriquez et al found obvious deterioration in K_max_ for control group of 2.21 D at 12 months [19].

With respect to visual acuity, changes between treated and control groups indicated remarkable differences for UCVA and BSCVA, which was -0.18 LogMAR and -0.10 LogMAR respectively. But it did not reach statistically significant improvement in UCVA. In 2012, Vinciguerra et al showed significant improvement of -0.21 LogMAR in UCVA and -0.19 LogMAR in BSCVA at 24 months [25]. There was also statistically significant reduction in manifest cylinder error after excluding the study of poor quality. However, non-significant difference was found in spherical equivalent, CCT and IOP. But two previous studies documented the reduction of spherical equivalent by 1.57 D at 24 months and 1.39 D at 48 months [25,26]. And one long term study in our analysis reported an mild increase of CCT value by 5.86 mm in the third year [15]. A similar review was recently published [27], which suggested that well-performed long-term RCTs and refinement in techniques were still needed to explore the potential benefit of CXL in slowing or reversing progression of keratoconus.

Adverse events were reported in the involved trials, but they were minimal and transient. Four studies observed that all treated eyes undergoing CXL exhibited some degree of corneal haze that resolved with time [15–17,20], one study by Hersh et al reported that more than 90% of eyes had the clinical appearance of stromal haze [18,28]. Six RCTs in a series of 179 treated eyes reported other complications as follows: one case of recurrent corneal erosion, one case of Descemet folds and corneal edema, one case of corneal edema associated with a paracentral infiltrate, two cases of sterile infiltrates, and one case of anterior chamber inflammation. One trial described 1 treatment failure after CXL with progression of +4.10 D in a patient who identified evidence of rosacea keratitis during the 36 months follow-up period [15]. In short, these adverse events were not serious and did not bring any impact on postoperative clinical outcomes. No intraoperative or serious postoperative complications were found in these studies. So, it may suggest that CXL is a safe procedure with few sight-threatening complications.

Certain limitations and biases remain in our study. First, each individual trial had a small sample size. We did not analyze other outcomes such as endothelial cell density or corneal biomechanics changes due to a lack of data. Second, the meta-analysis displayed some heterogeneity, suggesting the studies were not consistent in their conduct or varied at baseline. But stratification analysis and sensitivity analysis were conducted to explore the source of heterogeneity. Third, one study in our research offered CXL to control group after 6 months of follow-up. In the same study, baseline K_max_ in CXL group was steeper by 1.65 D than the control group (P = 0.052) [15]. This would lead us to underestimating the treatment effect of CXL and minimizing the progression in control group. Finally, most of the previously published studies are non-randomized interventional case series, only very few RCTs are available for cross-linking because of the lack of ethics of such studies. Thus, our meta-analysis compared only 6 studies with a very short follow-up except Wittig-Silva's one [15]. Investigation of the long-term continued stability and risks associated with the CXL procedure is essential.

Overall, our findings indicate that CXL procedure is safe and effective for the treatment of keratoconus, which results in significant reductions in corneal topographic measurements, manifest cylinder error, and improvement in visual outcomes. Further studies with long-term duration and larger sample size will be necessary to conclude in stabilization and absence of iatrogenicity for CXL.

## Supporting Information

S1 PRISMA Checklist Prisma checklist. (DOC)
